# Self-Aggregation, Antimicrobial Activity and Cytotoxicity of Ester-Bonded Gemini Quaternary Ammonium Salts: The Role of the Spacer

**DOI:** 10.3390/molecules28145469

**Published:** 2023-07-17

**Authors:** Yaqin Liang, Hui Li, Jiahui Ji, Jiayu Wang, Yujie Ji

**Affiliations:** Department of Chemistry, Changzhi University, Changzhi 046000, China; cy12006330@czc.edu.cn (H.L.); jjh2729320640@163.com (J.J.); 13799880798@163.com (J.W.); 15135410007@163.com (Y.J.)

**Keywords:** gemini surfactant, ester, antimicrobial activity, spacer, cytotoxicity

## Abstract

Five ester-bonded gemini quaternary ammonium surfactants C_12_-E_n_-C_12_ (n = 2, 4, 6), with a flexible spacer group, and C_12_-B_m_-C_12_ (m = 1, 2), with rigid benzene spacers, were synthesized via a two-step reaction and analyzed. Furthermore, the effects of the spacer structure, spacer length and polymerization degree on the self-aggregation, antimicrobial activity and cytotoxicity of C_12_-E_n_-C_12_ and C_12_-B_m_-C_12_ and their corresponding monomer *N*-dodecyl-*N*,*N*,*N*-trimethyl ammonium chloride DTAC were investigated. The results showed that C_12_-E_n_-C_12_ and C_12_-B_m_-C_12_ had markedly lower critical micellar concentration (CMC) values and lower surface tension than DTAC. Moreover, the CMC values of C_12_-E_n_-C_12_ and C_12_-B_m_-C_12_ decreased with increasing spacer length. In the case of equivalent chain length, the rigidity and steric hindrance of phenylene and 1,4-benzenediyl resulted in larger CMC values for C_12_-B_m_-C_12_ than for C_12_-E_n_-C_12_. The antibacterial ability of C_12_-E_n_-C_12_ and C_12_-B_m_-C_12_ was assessed using *Escherichia coli* (*E. coli*) and *Staphylococcus albus* (*S. aureus*) based on minimum inhibitory concentrations (MICs). Furthermore, C_12_-E_n_-C_12_ and C_12_-B_m_-C_12_ exhibited higher antimicrobial activity than DTAC and had stronger function toward *S. aureus* than *E. coli*. The antimicrobial activity was enhanced by increasing the spacer chain length and decreased with the increased rigidity of the spacers. The cytotoxic effects of C_12_-E_n_-C_12_ and C_12_-B_m_-C_12_ in cultured Hela cells were evaluated by the standard CCK8 method based on half-maximal inhibitory concentration (IC_50_). The cytotoxicity of C_12_-E_n_-C_12_ and C_12_-B_m_-C_12_ was significantly lower than alkanediyl-α,ω-bis(dimethyldodecylammonium) bromide surfactants and DTAC. The spacer structure and the spacer length could induce significant cytotoxic effects on Hela cells. These findings indicate that the five ester-bonded GQASs have stronger antibacterial activity and lower toxicity profile, and thus can be used in the pharmaceutical industry.

## 1. Introduction

Gemini quaternary ammonium surfactants (GQASs) contain two hydrophobic alkyl tails and two hydrophilic quaternary ammonium cations connected by a spacer at or near the head groups [1]. Many researchers have designed and synthesized GQASs with various structures by altering the spacer structure, spacer length, hydrophobic chain length and symmetry of the amphiphile molecule [2,3,4,5]. GQASs can form micelles and efficiently reduce surface tension. In addition, GQASs have better wetting ability as well as antimicrobial and anticorrosive properties as compared to their corresponding conventional single-chain surfactant counterparts [6]. Therefore, GQASs can be used in the biomedicine industry as antibacterial agents [7], corrosion inhibitors [8], drug carriers [9] and genetic vectors [10]. However, most GQASs are stable compounds with poor microbial and chemical degradability [11], posing risks to microorganisms in water and soil. GQASs can accumulate in soil and sediment and leak into underground water and wastewater, threatening human health and the Earth’s ecosystems [12,13]. Thus, the utilization of non-degradable GQASs has raised serious aquatic toxicity and environmental safety concerns, which has limited their application. As a result, some GQASs have been designed via different strategies to decrease environment pollution [13,14,15]. For example, biodegradable groups or cleavable bonds can be introduced in the GQAS structure to improve the environmental acceptability [15,16,17]. Enzymatic reaction/hydrolysis occurs in the presence of ester functional groups. Therefore, induction of ester groups in GQAS molecules may effectively improve their biodegradability and significantly reduce aquatic toxicity [18]. GQAS with ester groups have the mildness of surfactants containing ester groups and the high efficiency of quaternary ammonium surfactants [19]. Nazish et al. synthesized ester-bonded GQAS m-E_2_-m containing diester groups in the spacer tail and found that the cytotoxicity of the GQAS on 3T3-L1 fibroblast cells decreases with increasing alkyl carbon number [20]. GQAS with ester linkages also have special aggregation behavior because of the formation of intramolecular or intermolecular hydrogen bonds [15]. Tehrani-Bagha et al. also synthesized ester-bonded GQAS 12Q2OCO1Q12 and found that GQASs with ester groups in the spacer unit can easily biodegrade compared with GQASs with ester groups in the hydrophobic alkyl tails [21]. They also showed that 12Q2OCO1Q12 can be hydrolyzed to produce two products. Cytotoxic evaluation showed that the EC_50_ value of 12Q2OCO1Q12 is 0.27 mg·L^−1^, indicating that 12Q2OCO1Q12 is toxic to aquatic organisms [21]. Garcia et al. found that the aquatic toxicity of ester-based GQASs increases regularly with increasing hydrophobic alkyl tails [12]. Several studies have investigated the physicochemical properties, cytotoxicity and antimicrobial activity of GQASs with ester bonds based on changes in hydrophobic alkyl tails.

**Scheme 1:**
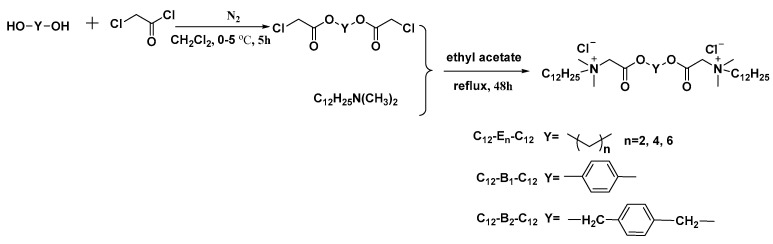
Synthesis of C_12_-E_n_-C_12_ and C_12_-B_m_-C_12_.

In view of the above points and the continuity of our previous works, GQASs with diester groups in the spacer and dodecyl alkyl chains, the same head groups and counterions were synthesized. They had different spacer structures and spacer lengths; the reaction scheme and abbreviations of the ester-bonded GQASs are shown in Scheme 1. The spacer of C_12_-E_n_-C_12_ (n = 2, 4, 6) contained a flexible spacer group, while the spacer of C_12_-B_m_-C_12_ (m = 1, 2) possessed a rigid benzene ring. The self-aggregation, antimicrobial activity and cytotoxicity of ester-bonded GQASs and their corresponding monomer *N*-dodecyl-*N*,*N*,*N*-trimethyl ammonium chloride DTAC were also surveyed. Furthermore, the effects of spacer length, spacer structure and polymerization degree on the properties of C_12_-E_n_-C_12_ and C_12_-B_m_-C_12_ were evaluated. Therefore, this study establishes the structure–property relationship of ester-bonded GQAS, providing the basis for the development of biocompatible and nontoxic gemini surfactants with high antimicrobial activity.

## 2. Results and Discussion

### 2.1. Surface Tension

**Figure 1:**
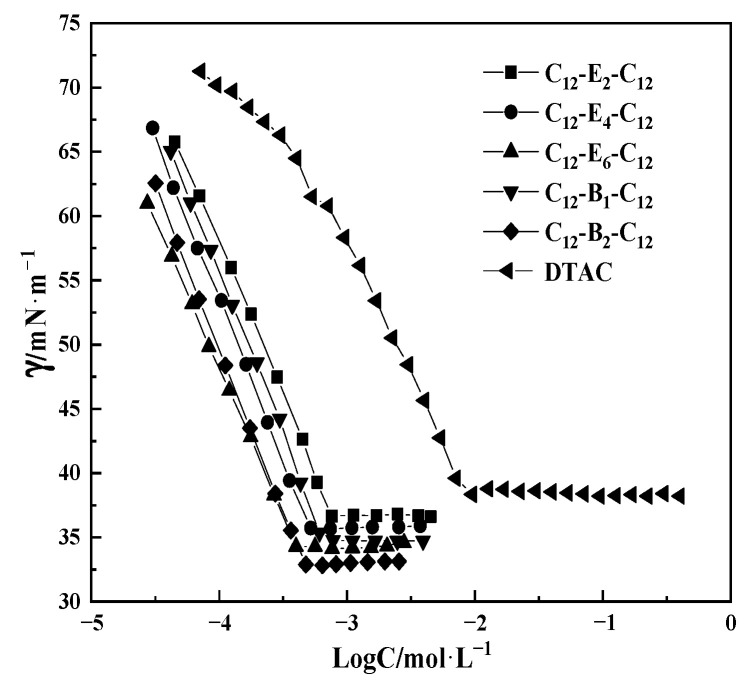
Surface tension as function of the log C for ester-bonded GQASs and DTAC at 25 °C.

Figure 1 illustrates the surface tension (γ) curves for the synthesized ester-bonded GQASs and DTAC as a function of logarithm concentration at 25 °C. The surface tension values noticeably decreased and then stabilized after a certain concentration, corresponding to CMC. The CMC and surface parameters of the synthesized ester-bonded GQASs and DTAC were determined from the γ-logC curves, which are listed in Table 1.

As shown in Table 1, the observed CMC values of C_12_-E_2_-C_12_, C_12_-E_4_-C_12_, C_12_-E_6_-C_12_, C_12_-B_1_-C_12_ and C_12_-B_2_-C_12_ were 0.815, 0.528, 0.378, 0.618 and 0.467 mmol·L^−1^, respectively, which were significantly lower than that of DTAC (10.25 mmol·L^−1^). This difference could be due to the presence of two hydrophobic alkyl tails and two hydrophilic quaternary ammonium cations at the molecular structures of the ester-bonded GQASs, which enhanced the hydrophobic synergism among the hydrophobic alkyl tails [22]. The additional hydration in the spacer overcomes the Coulombic repulsive forces between the head group quaternary ammonium cations [23]. The ester groups in the spacer for the ester-bonded GQASs, which are more polar than the methylene spacers, promoted water-mediated hydrogen bonding between ester groups.

For the ester-bonded GQASs, the CMC values followed the order C_12_-E_4_-C_12_ < C_12_-B_1_-C_12_ < C_12_-E_6_-C_12_ < C_12_-B_2_-C_12_ < C_12_-E_2_-C_12_. Furthermore, C_12_-E_2_-C_12_ had the highest CMC value among C_12_-E_n_-C_12_. Two head group quaternary ammonium cations in C_12_-E_2_-C_12_ were connected with ethylene: two ester and two methylene groups. The spacer group was constrained, thus remaining in extended conformation and lying prone at the micelle–water (or air–water) interface [22,23,24,25], which limited the micellization process. The hydrophobicity and flexibility of C_12_-E_n_-C_12_ increased with the increasing number of n of the polymethylene spacer from 2 to 6. The loop orientation of the longer spacer was extended toward the hydrophobic micellar core, and the spacers adopted the folder conformation toward the air side, preventing the hydration of the micelle. As a result, the CMC values decreased compared with n = 2 and n = 4 to 6. Zana also studied a series of GQASs with 12-s-12 with hydrophilic flexible spacers (s represents the number of –CH_2_ groups in the spacer tail) and found that CMC values increased up to s = 6 and then decreased for s = 8, 10, 12 [26,27]. C_12_-B_2_-C_12_ and C_12_-B_1_-C_12_ had larger CMC values than C_12_-E_6_-C_12_ and C_12_-E_4_-C_12_, respectively, indicating that the rigidity of the spacer influenced the micellar aggregation [27]. According to the report by Zhu et al., the contribution of the benzene ring to the molecular structure of surfactants in the surface properties is similar to that of 3.5–4.0 methylene units [27]. We choose C_12_-E_4_-C_12_ and C_12_-B_1_-C_12_ as examples for discussion. The spacer length of C_12_-B_1_-C_12_ is almost equal to that of C_12_-E_4_-C_12_. Therefore, the spacers of C_12_-E_6_-C_12_ and C_12_-E_4_-C_12_ had n-hexanediyl and n-butanediyl units, respectively. The flexibility of these units allowed the spacers to easily adopt looped conformation on the air–water interface. C_12_-B_2_-C_12_ and C_12_-B_1_-C_12_ had phenylene and 1,4-benzenediyl units, and the benzene ring in the spacer was rigid and large in volume, forming the steric inhibition of C_12_-B_2_-C_12_ and C_12_-B_1_-C_12_ caused by the rigid spacer [28]. The rigidity and steric hindrance of phenylene and 1,4-benzenediyl can prevent its incorporation in the hydrophobic core of the micelle. This would inhibit micelle formation and lead to larger CMC values. Regarding the aggregation behavior of C_12_-B_m_-C_12_, C_12_-B_2_-C_12_ had a stronger tendency to aggregate than C_12_-B_1_-C_12_. Such behavior could be attributed to the rigid spacer of C_12_-B_m_-C_12_. The rigid hydrophobic spacer of C_12_-B_1_-C_12_ lay flat at the surface at the micelle–water (or air–water) interface; the spacer of C_12_-B_2_-C_12_ could be formed by adding two methylene groups to the spacer of C_12_-B_1_-C_12_, which increased hydrophobic interaction and favored micellization.

In this study, γ_CMC_ was defined as the effectiveness of the ester-bonded GQASs or DTAC in reducing the surface tension of water. The γ_CMC_ values of C_12_-E_2_-C_12_, C_12_-E_4_-C_12_, C_12_-E_6_-C_12_, C_12_-B_1_-C_12_ and C_12_-B_2_-C_12_ were 36.92, 35.58, 34.29, 34.58 and 32.82 mN·m^−1^, respectively, which were slightly lower than that of DTAC (38.57 mN·m^−1^). This indicates C_12_-B_m_-C_12_ and C_12_-E_n_-C_12_ are adsorbed strongly at the air–water interface and are effective in reducing the surface tension of water.

The minimum surface area (*A_min_*) occupied per the synthesized ester-bonded GQASs or DTAC molecule and the surface excess concentration (*Γ_max_*) were estimated based on the following Gibbs equations, Equations (1) and (2) [6]:

(1) $$\Gamma_{\max}=-\frac{1}{2.303nRT}{\left(\frac{d\gamma }{d\log C}\right)}_{T}$$

(2) $$A_{\min}=\frac{1}{N_{A}\Gamma_{\max}}$$

where *R* = 8.314 J·mol^−1^·K^−1^, *T* = 298.15 K, and *N_A_* = 6.023 × 10^23^ mol^−1^; *γ* and *C* represent the surface tensions and the concentration of ester-bonded GQASs or DTAC, respectively; *dγ*/*dlogC* is the slope of the line before the CMC in the γ-lgC curves. The parameter n is a constant corresponding to the number of individual ions for the surfactants adsorbed at the interface. *n* is taken as 3 for the ester-bonded GQASs, implying the ion paring and dissociation of two univalent counterions from the dicationic quaternary ammonium headgroups, while *n* is taken as 2 for DTAC, implying the surfactant ion and the chloridion counterion were monovalent.

As shown in Table 1, the *A_min_* values of C_12_-E_2_-C_12_, C_12_-E_4_-C_12_, C_12_-E_6_-C_12_, C_12_-B_1_-C_12_, C_12_-B_2_-C_12_ and DTAC were 1.82, 1.65, 1.42, 1.77, 1.58 and 0.72 nm^2^, respectively. The ester-bonded GQASs had larger *A_min_* values than DTAC, indicating the impact of the polymerization degree on the *A_min_* value. C_12_-E_2_-C_12_ had the highest *A_min_* value among C_12_-E_n_-C_12_, demonstrating that the spacer length impacts the *A_mi_*_n_ value. The flexibility and hydrophobicity of C_12_-E_n_-C_12_ increases because of the lengthening of the spacer, and the spacer adopted the folder conformation to the water side of the air–water interface, thus decreasing *A_min_* value and increasing the aggregation density of surfactant per unit area. As a result, the *Γ_max_* value increased with increasing the spacer length. Moreover, C_12_-B_2_-C_12_ and C_12_-B_1_-C_12_, with the rigid benzene ring in the spacer, had a larger *A_min_* value than C_12_-E_6_-C_12_ and C_12_-E_4_-C_12_, with a similar length of flexible spacer, respectively, indicating that the spacer structure can impact the *A_min_* value. Compared with the flexible spacer of C_12_-E_6_-C_12_ and C_12_-E_4_-C_12_, the benzene ring of C_12_-B_2_-C_12_ and C_12_-B_1_-C_12_ increased the spacer rigidity, thus hindering the conformational change of C_12_-B_m_-C_12_ at the air–water interface. The same change tendency for the spacer rigidity with regard to the *A_min_* value was reported by Zheng [23]. Zheng et al. found that the gemini surfactant (Mor)m-P-m containing the benzene ring as the spacer had a larger *A_min_* value, and the spacer tended to lay on the air–water interface [23]. However, introducing two flexible methylene units to the spacer of C_12_-B_1_-C_12_ reduced the *A_min_* value from 1.77 nm^2^ to 1.58 nm^2^ since the spacer of C_12_-B_2_-C_12_ became more flexible and more hydrophobic.

The adsorption efficiency pC_20_ refers to the negative logarithm concentration of the ester-bonded GQASs and DTAC to decrease the surface tension of the aqueous system by 20 mN·m^−1^ [23]. Higher pC_20_ values indicate greater efficiency in reducing surface tension. Table 1 shows that the pC_20_ values of C_12_-E_2_-C_12_, C_12_-E_4_-C_12_, C_12_-E_6_-C_12_, C_12_-B_1_-C_12_, C_12_-B_2_-C_12_ and DTAC are 3.07, 3.19, 3.36, 3.15, 3.28 and 2.95, respectively. Obviously, the pC_20_ values of ester-bonded GQASs are higher than that of DTAC, suggesting that the ester-bonded GQASs are strongly adsorbed at the water–air interface and have greater efficiency in reducing surface tension. The pC_20_ values for C_12_-E_n_-C_12_ displayed an increasing trend from 3.07 to 3.36 with spacer lengthening. The pC_20_ value of C_12_-B_2_-C_12_ was larger than that of C_12_-B_1_-C_12_, which may be associated with the arrangement of the spacer at the water–air interface.

### 2.2. Conductivity Measurements

**Figure 2:**
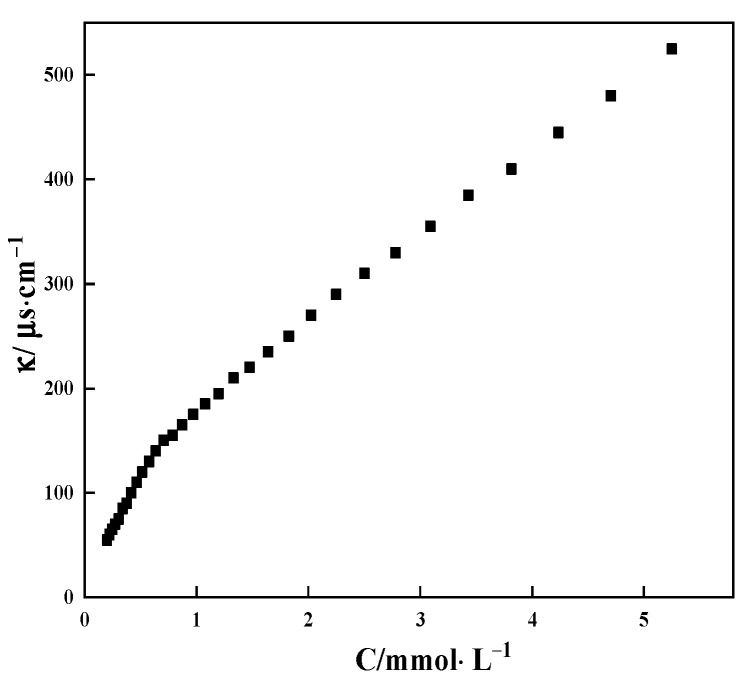
Variation in specific conductivity (ĸ) with concentration C of C_12_-E_2_-C_12_ at 25 °C.

The aggregation behavior of the ester-bonded GQASs or DTAC in aqueous solutions was investigated by the conductivity method at 25 °C. The representative conducto-metric profiles for C_12_-E_2_-C_12_ at 25 °C are shown in Figure 2. The plots of κ vs. C for the other four ester-bonded GQASs and DTAC are provided in Figures S17–S21 in the Supplementary Materials. The CMC values were determined by the inflection point of κ vs C and are presented in Table 1. The CMC values for C_12_-E_2_-C_12_, C_12_-E_4_-C_12_, C_12_-E_6_-C_12_, C_12_-B_1_-C_12_, C_12_-B_2_-C_12_ and DTAC were 0.985, 0.643, 0.489, 0.745, 0.579 and 11.88 mmol·L^−1^, respectively, which were slightly higher than those investigated by surface tension measurements. However, the CMC values determined using the conductivity method and the surface tension measurements followed the same tendency. The degree of counterion binding (β), the ability of chloride counterions to bind to the stern layer of the micelle, was determined from the slope values before and after the CMC obtained by the conductivity plot. Table 1 shows that the β values for C_12_-E_2_-C_12_, C_12_-E_4_-C_12_, C_12_-E_6_-C_12_, C_12_-B_1_-C_12_, C_12_-B_2_-C_12_ and DTAC are 0.62, 0.51, 0.45, 0.49, 0.58 and 0.66, respectively, and the β values of ester- bonded GQASs are all slightly lower than that of DTAC, possibly because of the charge-density of the head group polarity [6]. The ester-bonded GQASs had larger *A_min_* values compared to DTAC because of the presence of the spacer, thus affecting binding between chloride counterions and micelles. The β values of the C_12_-E_n_-C_12_ series decreased with an increase in the spacer chain length. Zhang and Hou et al. reported similar findings for different structures of cationic gemini surfactants [22,24]. Furthermore, the β values for C_12_-B_1_-C_12_ and C_12_-B_2_-C_12_ were lower than those of C_12_-E_4_-C_12_ and C_12_-E_6_-C_12_, respectively. Martin et al. also reported similar findings for alkanediyl-α-ω-bis (dodecyl-dimethylammonium) bromide [25]. The *A_min_* values of C_12_-B_1_-C_12_ and C_12_-B_2_-C_12_ on the air–water interface were larger than those of C_12_-E_4_-C_12_ and C_12_-E_6_-C_12_. Rigidity and steric hindrance of phenylene and 1,4-benzenediyl of C_12_-B_1_-C_12_ and C_12_-B_2_-C_12_ could prevent incorporation in the nonpolar region of the micellar core [28]. Moreover, the rigid spacer of C_12_-B_1_-C_12_ and C_12_-B_2_-C_12_ hindered the binding between chloride counterions and micelles [28].

The Gibbs free energy of micellization, $\Delta G_{m}^{\theta }$, of the synthesized ester-bonded GQASs or DTAC can be computed using Equation (3) below [6].

(3) $$\Delta G_{m}^{\theta }=RT(1/2+\beta )\ln X_{CMC}$$

where *X_CMC_* = *CMC*/55.4; 55.4 is derived from 1 L of water corresponding to 55.4 mol of water at 25 °C [25]. Table 1 shows that the $\Delta G_{m}^{\theta }$ values for C_12_-E_2_-C_12_, C_12_-E_4_-C_12_, C_12_-E_6_-C_12_, C_12_-B_1_-C_12_, C_12_-B_2_-C_12_ and DTAC are −25.97, −28.20, −32.22, −27.30, −30.61 and −22.78 kJ·mol^−1^, respectively. Notably, all $\Delta G_{m}^{\theta }$ values of the ester-bonded GQASs and DTAC are negative, which indicates that the micellization is an energy-favorable process. The $\Delta G_{m}^{\theta }$ values of the ester-bonded GQASs were more negative than that of DTAC, indicating that the ester-bonded GQASs were more favored to form micelles. Moreover, the $\Delta G_{m}^{\theta }$ values decreased with the elongation of the spacer length for C_12_-E_n_-C_12_. The $\Delta G_{m}^{\theta }$ values of C_12_-B_1_-C_12_ and C_12_-B_2_-C_12_ with rigid spacers were higher than those of C_12_-E_4_-C_12_ and C_12_-E_6_-C_12_, respectively, demonstrating that the spacer rigidity had an effect on the micellization.

### 2.3. Antimicrobial Activity

The antibacterial effect of the ester-bonded GQASs and DTAC was assessed using *S. aureus* and *E. coli.* The minimum inhibitory concentrations (MICs) were examined by the agar dilution method, and these data are listed in Table 2. It was found that the five ester-bonded GQASs and DTAC exhibited stronger antibacterial activity toward *S. aureus* than *E. coli*, probably due to the different cell membrane structures of *S. aureus* and *E. coli*. The cell walls of *S. aureus* consist of many layers of peptidoglycan and teichoic acids, promoting the permeation of surfactants through the phospholipid layer of the cell membrane. However, the cell walls of *E. coli* contain a thin layer of peptidoglycan, lipopolysaccharides and porins, inhibiting the permeation of surfactants and the entrance of the hydrophobic alkyl tails of surfactants [22]. The MIC values of the five synthesized ester-bonded GQASs ranged from 13 to 22 μg·mL^−1^ for *E. coli* and from 12 to 5 μg·mL^−1^ for *S. aureus*, while the MIC values of DTAC were 68 μg·mL^−1^ for *E. coli* and 16 μg·mL^−1^ for *S. aureus.* Notably, the MIC values of the five ester-bonded GQASs on both *E. coli* and *S. aureus* were smaller than that of DTAC, indicating that the ester-bonded GQASs exhibited a stronger antimicrobial effect than DTAC. The antibacterial mechanism of cationic surfactants is induced by the electrostatic interaction between the positively charged headgroup of surfactants and the negatively charged phospholipid surface of the bacterial cell wall. In addition, hydrocarbon tails of cationic surfactants can penetrate and disturb the selective permeability of the membrane, leading to the leakage of cell contents and, ultimately, cell death [22,23]. The ester-bonded GQASs contained organic quaternary ammonium cations and two hydrophobic dodecyl chains in the molecule, facilitating stronger electrostatic and hydrophobic interaction with cell membranes than DTAC.

Amongst the C_12_-E_n_-C_12_ series examined, the MIC values gradually decreased for both *S. aureus* and *E. coli* with increasing of the spacer chain length. Similar behavior has been observed for MIC values of different molecular structures of GQASs [9,22,23]. The increase in spacer length resulted in increasing hydrophobicity and flexibility, thus promoting the adsorption tendency of C_12_-E_n_-C_12_ molecules at cell membrane surfaces. Meanwhile, the β values of C_12_-E_n_-C_12_ decreased with the spacer chain lengthening due to the decrease of the charge density. This findings indicate that C_12_-E_6_-C_12_ may have a weaker electrostatic interaction with the cell membrane than C_12_-E_4_-C_12_ and C_12_-E_2_-C_12_. The joint effects of electrostatic and hydrophobic interactions with cells made the MIC values of C_12_-E_n_-C_12_ follow the pattern C_12_-E_2_-C_12_ > C_12_-E_4_-C_12_ > C_12_-E_6_-C_12_, meaning that C_12_-E_6_-C_12_ has the highest antimicrobial activity on *S. aureus* and *E. coli*. Nonetheless, the MIC values of C_12_-E_6_-C_12_ and C_12_-E_4_-C_12_ on *S. aureus* and *E. coli* were lower than those of C_12_-B_2_-C_12_ and C_12_-B_1_-C_12_, indicating that C_12_-E_6_-C_12_ and C_12_-E_4_-C_12_ have stronger electrostatic and hydrophobic interactions with the cell wall and better antibacterial properties. This could be due to the spacer of the ester-bonded GQASs. C_12_-B_m_-C_12_ with phenylene and 1,4-benzenediyl as the spacer had a more steric effect than C_12_-E_6_-C_12_ and C_12_-E_4_-C_12_ with n-hexanediyl and n-butanediyl as the spacer, thus reducing the permeability of the cell membrane. In addition, conformational changes of C_12_-B_2_-C_12_ and C_12_-B_1_-C_12_ can weaken the hydrophobic binding between the tails and the lipid layers of cells [21].

### 2.4. Cytotoxicity of Gemini Surfactants

**Figure 3:**
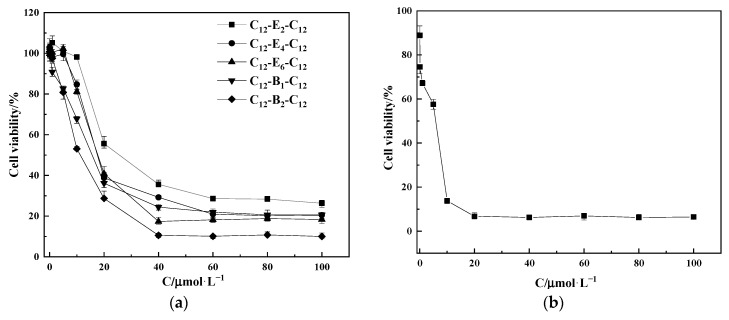
Concentration–viability curves of the Hela cells exposed to the ester-bonded GQASs and DTAC: (**a**) the ester-bonded GQASs; (**b**) DTAC.

The cytotoxicity profiles of ester-bonded GQASs and DTAC in cultured Hela cells were assessed by the standard CCK8 method. The value of half-maximal inhibitory concentration (IC_50_) was quantified based on the profiles of the cell viability versus surfactant concentration (*C*) (Figure 3), and the results are shown in Table 3. The IC_50_ values were 14.55 and 12.75 μmol·L^−1^ for C_12_-B_m_-C_12_ (m = 1, 2), respectively, and ranged from 36.24 to 21.01 μmol·L^−1^ for C_12_-E_n_-C_12_ with n from 2 to 6. The IC_50_ values of ester-bonded GQASs were higher than that of DTAC, indicating that the ester-bonded GQASs are less toxic than DTAC. This was in accordance with the findings of Garcia [12]. In comparison with the traditional GQASs, the IC_50_ values of ester-bonded GQASs were greater than those of 12-s-12, in the range from 2.8 to 3.1 μmol·L [22]. This indicated that the ester-bonded GQASs have lower cytotoxicity compared to those reported for the traditional GQASs, and the ester group within the spacer functionality can reduce their cytotoxicity, which was slightly analogous to the study by Pinazo with GQASs containing ester bonds in their spacers [29]. This is because the introduction of ester to GQASs can enhance the hydrophilicity; meanwhiles the ester bond linking the head group to the two hydrophobic chains had lower chemical stability and could be easily hydrolyzed, giving rise to non-toxic species [30,31]. Moreover, the IC_50_ values of C_12_-E_n_- C_12_ and C_12_-B_m_-C_12_ decreased with the growth of the spacer chain length; then, the cytotoxicity increased slightly with elongation of spacer chain, which was in agreement with the data available on cytotoxicity activity of other gemini surfactants [31]. The IC_50_ values of C_12_-B_1_-C_12_ and C_12_-B_2_-C_12_ were lower than those of C_12_-E_4_-C_12_ and C_12_-E_6_-C_12_, respectively, which showed that the structure of the spacer had an effect on the cytotoxicity activity of ester-bonded GQASs, which could be attributed to the combined effects of electrostatic and hydrophobic interactions of ester-bonded GQASs with Hela cells [21].

## 3. Materials and Methods

### 3.1. Materials

1,4-benzene-dimethanol (99%), hydroquinone (99.5%), *N*,*N*-dimethyldodecyl amine (95%), ethylene glycol (99%), hexane-1,6-diol (99%), butane-1,4-diol (99%) and DTAC were sourced from Aladdin Industrial Corporation. Chloroacetyl chloride was obtained from Xiya Reagent Factory. Beef-extract paste, agar powder and protein peptone were purchased from Sinopharm Reagent Co., Ltd. Bovine serum, streptomycin and penicillin were sourced from Sigma-Aldrich. *E. coli* and *S. aureus* were provided by Shanghai Microspectrum Chemical Technology Service Co., Ltd. Human cervical squamous cell carcinoma Hela cells was provided by the Department of Life Science, Changzhi University. All other reagents were of analytical grade. Milli-Q water, obtained by sub-boiling distilled water, was used for all experimental procedures.

### 3.2. Synthesis and Characterization

An ALPHA infrared spectrophotometer (Bruker BioSpin Co., Ettlingen, Germany) was used to study the FTIR spectra of the synthetic GQAS. Meanwhile, ^1^H NMR spectra and ^13^C NMR spectra were determined using a BRUKER Ascend^TM^ 400 instrument (Bruker BioSpin Co., Ettlingen, Germany). A Compact mass spectrometer (Bruker Co., Bremen, Germany) was used to detect the mass spectra of ester-based GQASs using ESI as an ion source. A K100 tensiometer (Krüss Co., Hamburg, Germany) was used to determine surface tensions of the synthetic GQASs and DTAC, while a DDSJ-308F conductimeter (Shanghai Rex Electric Chemical Co., Shanghai, China) was used to determine the conductivity. A microplate reader (Synergy H1, Agilent BioTek, Santa Clara, CA, USA) was used to evaluate the cytotoxicity of the surfactants.

Take C_12_-E_2_-C_12_ as an example for demonstrating the procedure of synthesis. Freshly distilled triethylamine (80 mmol, 8.52 g) as an acid-binding agent and ethylene glycol (30 mmol, 1.88 g) were added to 100 mL absolute dichloromethane as a solvent at room temperature and in a nitrogen atmosphere. Chloroacetyl chloride (90 mmol, 10.16 g) dissolved in 40 mL dichloromethane was added to the mixed solution dropwise at 0–5 °C for 5 h. The viscous liquid was achieved by washing with 0.5 mol·L^−1^ Na_2_CO_3_ aqueous solution, drying with anhydrous MgSO_4_, suction filtering, discolorating with active carbon and evaporating the solvent.

The obtained product (20 mmol, 3.72 g) was dissolved in 40 mL anhydrous ethyl acetate, followed by addition of 50 mmol 10.67 g *N*,*N*-dimethyldodecylamine under even stirring. The solution was refluxed for 48 h, cooled down to ambient temperature and filtered. The wet filter cake was recrystallized thrice using acetone and ethanol (1:1) to obtaining the product as fairy white powder.

*Ethane-1,2-diyl bis(N,N-dimethyl-N-dodecylammonium acetoxy) dichloride (C_12_-E_2_-C_12_)*. 9.71 g, 85% yield. FT-IR (KBr, cm^−1^): 2915, 2847, 1738, 1047, 720. ^1^H NMR (DMSO, ppm): 0.84–0.88 (t, *J* = 8.0 Hz, 6H), 1.25–1.28 (m, 36H), 1.66–1.73 (m, 4H), 3.25 (s, 12H), 3.51–3.55 (t, *J* = 8.0 Hz, 4H), 4.45 (s, 4H), 4.70 (s, 4H). ^13^C NMR (DMSO, ppm): 14.42, 22.25, 22.56, 26.19, 28.94, 29.18, 29.27, 29.42, 29.49, 31.60, 51.16, 60.92, 63.42, 64.93, 165.01. HRMS(ESI+): m/z calcd for [M-Cl]^2+^/2: 285.2663, found: 285.2679.

*Butane-1,4-diyl bis(N,N-dimethyl-N-dodecylammonium acetoxy) dichloride (C_12_-E_4_ -C_12_)*. 9.96 g, 83% yield. FT-IR (KBr, cm^−1^): 2920, 2850, 1767, 1050, 718. ^1^H NMR (DMSO, ppm): 0.84–0.88 (t, *J* = 8.0 Hz, 6H), 1.25–1.28 (m, 36H), 1.66–1.72 (m, 8H), 3.23 (s, 12H), 3.49–3.53 (t, *J* = 8.0 Hz, 4H), 4.20–4.23 (s, 4H), 4.56 (t, 4H). ^13^C NMR (DMSO, ppm): 14.58, 22.18, 22.56, 25.21, 26.26, 28.18, 28.90, 29.18, 29.21, 29.39, 29.48, 31.76, 51.28, 61.14, 64.75, 66.15, 165.37. HRMS(ESI+): m/z calculated for [M-Cl]^2+^/2: 299.2819, found: 299.2844.

*Hexane-1,6-diyl bis(N,N-dimethyl-N-dodecylammonium acetoxy) dichloride (C_12_-E_6_-C_12_)*. 10.53 g, 84% yield. FT-IR (KBr, cm^−1^): 2913, 2850, 1746, 1062, 719. ^1^H NMR (DMSO, ppm): 0.80–0.87 (t, *J* = 8.0 Hz, 6H), 1.25–1.37 (m, 40H), 1.61–1.71 (m, 8H), 3.20–3.24 (s, 12H), 3.50–3.55 (t, *J* = 8.0 Hz, 4H), 4.16–4.20 (t, *J* = 8.0 Hz, 4H), 4.60 (s, 4H). ^13^C NMR (DMSO, ppm): 14.42, 22.23, 22.56, 24.82, 26.14, 28.91, 29.18, 29.23, 29.40, 29.48, 31.76, 51.35, 60.95, 64.74, 65.64, 165.37. HRMS(ESI+): m/z calculated for [M-Cl]^2+^/2: 313.2976, found: 313.2995.

*Benzene-1,4-diyl bis(N,N-dimethyl-N-dodecylammonium acetoxy) dichloride (C_12_-B_1_-C_12_)*. 10.65 g, 86% yield. FT-IR (KBr, cm^−1^): 2915, 2847, 1738, 1466, 1414, 720. ^1^H NMR (DMSO, ppm): 0.85–0.88 (t, *J* = 8.0 Hz, 6H), 1.26–1.31 (m, 36H), 1.74–1.78 (m, 4H), 3.23 (s, 12H), 3.53–3.57 (t, *J* = 8.0 Hz, 4H), 4.70–4.79 (s, 4H), 7.39 (m, 4H). ^13^C NMR (DMSO, ppm): 14.42, 22.27, 22.56, 26.12, 28.91, 29.17, 29.22, 29.47, 31.76, 51.48, 62.26, 65.29, 123.50, 147.62, 164.32. HRMS(ESI+): m/z calculated for [M-Cl]^2+^/2: 309.2663, found: 309.2679.

*Phenylene-1,2-diyl bis(N,N-dimethyl-N-dodecylammonium acetoxy) dichloride (C_12_-B_2_-C_12_)*. 10.61 g, 82% yield. FT-IR (KBr, cm^−1^): 2916, 2850, 1740, 1467, 1415, 722. ^1^H NMR (DMSO, ppm): 0.87–0.92 (t, *J* = 8.0 Hz, 6H), 1.27–1.34(m, 36H), 1.72 (m, 4H), 3.55 (s, 12H), 3.84 (t, *J* = 8.0 Hz, 4H), 5.16 (s, 4H), 5.33(m, 4H), 7.35 (m, 4H). ^13^C NMR (DMSO, ppm): 14.42, 22.37, 22.66, 26.42, 29.01, 29.22, 29.32, 29.42, 29.57, 31.88, 51.60, 61.34, 65.37, 123.38, 147.91, 164.24. HRMS(ESI+): m/z calculated for [M-Cl]^2+^/2: 323.2819, found: 323.2808.

### 3.3. Surface Tension Measurement

A K100 automatic tensiometer was used to determine the surface tension of the ester-bonded GQASs and DTAC at the water–air interface with a Wilhelmy plate at 25 °C. Three consecutive readings with a standard deviation less than ±0.2 mN·m^−1^ were recorded at each concentration. The surface tensions values were plotted against the logarithm of the surfactant concentration. The CMC and γ_CMC_ values of the ester-bonded GQASs and DTAC were determined based on the inflection point on the plot of γ against log C [6].

### 3.4. Conductivity Measurements

Conductivity of the synthesized ester-bonded GQAS and DTAC solutions was determined using a conductimeter equipped with platinum electrodes in conjunction with a conductivity cell with a cell constant of 0.98 cm^−1^. The surfactant solutions were placed in thermostatic water bath at 25 °C, maintained using a temperature controller. The uncertainty of the conductivity measurements was ±0.2 μs·cm^−1^ [6]. The CMC values were determined based on the intersection between two line segments of the plots [6]. The conductivity was measured three times for each solution.

### 3.5. Antimicrobial Activity

Antimicrobial activity of the ester-bonded GQASs and DTAC against gram-positive (*S. aureus*) bacterium and gram-negative (*E. coli*) bacterium was evaluated by the broth dilution method [21]. The bacterial strains were each cultured in the nutrient agar plates with an inoculating loop at 37 °C overnight, then diluted to about 10^7^ CFU·mL^−1^. A total of 1 mL of the surfactant solution was added to the nutrient broths, followed by inoculation of 10 mL of bacterial suspension at 37 °C for 1 h [1]. The test organisms were coated on the Petri dishes by the tenfold dilution method, then inoculated at 37 °C for 24 h. The bacterial liquid without any surfactant solution was used as the blank control group. The minimum inhibitory concentration (MIC) values were quantified according to the concentration of the surfactant needed to achieve a sterilization rate of 90% [7]. All tests were performed in triplicate.

### 3.6. Cytotoxicity Assay (CCK-8 Test)

The cytotoxicity of the synthesized ester-bonded GQASs and DTAC in Hela cells was tested using the CCK-8 assay, as previously reported [32,33]. Hela cells (100 μL, 4×10^3^ per cell in a 96-well plate) were treated with 100 μL culture medium per well and grown in an incubator for 24 h with a temperature of 37 °C and 5% CO_2_ [33]. Hela cells were treated with different concentrations of the ester-bonded GQASs or DTAC (0.0, 0.05, 0.1, 1, 5, 10, 20, 40, 60, 80 and 100 μmol·L^−1^) overnight. A total of 10 μL of CCK8 solution was added to each well in 96-well plate (away from light) [23]. After 4 h, CCK-8–containing medium was shaken for 15 min. The absorption was then read at 450 nm with a microplate reader. The cell viability of the ester-bonded GQASs and DTAC was calculated according to the following equation:

(4) $$Cell\;viablity/\%=\frac{A-A_{0}}{A_{c}^{}-A_{0}}\times 100\%$$

*A*, *A*_0_ and *A_c_* represent the absorbance (450 nm) in the treated cells, the blank in which the cells almost completely died, and the untreated cells, respectively [22]. The half maximal inhibitory concentration (IC_50_) was quantified based on the plot of the cell viability versus the logarithm of surfactant concentration (*C*). All the data were expressed as mean ± standard deviations for six different experiments.

## 4. Conclusions

In summary, a series of ester-bonded GQASs with different spacer groups were synthesized, and their self-aggregation, antimicrobial activity and cytotoxicity were systematically studied. The CMC values of C_12_-E_n_-C_12_ and C_12_-B_m_-C_12_ were found to be between 0.895 and 0.597 μmol·L^−1^, which was markedly lower compared with DTAC. The CMC values of C_12_-E_n_-C_12_ and C_12_-B_m_-C_12_ decreased with the spacer lengthening in the series of homologs. In case of the equivalent chain length, C_12_-B_m_-C_12_ with the rigid spacer exhibited higher CMC values than C_12_-E_n_-C_12_ containing the flexible spacer. C_12_-E_n_-C_12_ and C_12_-B_m_-C_12_ exhibited stronger antibacterial activity than DTAC, and they showed greater inhibition of *S. aureus* than *E. coli*. The MIC values of C_12_-E_n_-C_12_ and C_12_-B_m_-C_12_ were found to decrease with the n changing from 2 to 6 and the m changing from 1 to 2. They decreased with the decrease in rigidity of the spacers. The cytotoxicity evaluation of C_12_-E_n_-C_12_ and C_12_-B_m_-C_12_ in cultured Hela cells showed that the cytotoxicity of C_12_-E_n_-C_12_ and C_12_-B_m_-C_12_ was obviously lower than alkanediyl-α,ω-bis (dimethyldodecyl ammonium) bromide surfactants and DTAC. The spacer structure and the spacer length could induce significant cytotoxic effects on Hela cells. Therefore, this study offers deep insight into the interfacial properties, the antibacterial activity and the cytotoxicity of the ester-bonded GQASs and provides ideas for the potential technical and biological applications.

## Figures and Tables

**Table 1:** The surface activities of the ester-bonded GQASs and DTAC at 25 °C.

Surfactant	CMC ^1^ (mmol·L^−1^)	CMC ^2^ (mmol·L^−1^)	β	γ_CMC_ (mN·m^−1^)	*Γ_max_* × 10^6^ (mol·m^−2^)	*A_min_* (nm^2^)	pC_20_	$\Delta G_{m}^{\theta }$ (kJ·mol^−1^)
C_12_-E_2_-C_12_	0.815	0.895	0.62	36.92	0.91	1.82	3.07	−25.97
C_12_-E_4_-C_12_	0.528	0.706	0.51	35.58	1.01	1.65	3.19	−28.20
C_12_-E_6_-C_12_	0.378	0.502	0.45	34.29	1.17	1.42	3.36	−32.22
C_12_-B_1_-C_12_	0.618	0.813	0.49	34.58	0.94	1.77	3.15	−27.30
C_12_-B_2_-C_12_	0.467	0.597	0.58	32.82	1.05	1.58	3.28	−30.61
DTAC	10.25	11.88	0.66	38.57	2.32	0.72	2.95	−22.78

^1^ Surface tension method. ^2^ Electrical conductivity method.

**Table 2:** The MIC values of the ester-bonded GQASs and DTAC against *E. coli* and *S. aureus* (μg·mL^−1^).

Strains	C_12_-E_2_-C_12_	C_12_-E_4_-C_12_	C_12_-E_6_-C_12_	C_12_-B_1_-C_12_	C_12_-B_2_-C_12_	DTAC
*S. aureus*	12	10	5	11	8	16
*E. coli*	22	15	13	19	17	68

**Table 3:** Cytotoxic effect (IC_50_) of the ester-bonded GQASs and DTAC on Hela cells.

Surfactants	C_12_-E_2_-C_12_	C_12_-E_4_-C_12_	C_12_-E_6_-C_12_	C_12_-B_1_-C_12_	C_12_-B_2_-C_12_	DTAC
IC_50_ (μmol·L^−1^)	36.34 ± 2.04	29.36 ± 3.11	21.01 ± 2.11	14.55 ± 1.23	12.75 ± 2.45	5.04 ± 1.23

## Data Availability

Not applicable.

## Supplementary Materials

The following supporting information can be downloaded at: https://www.mdpi.com/article/10.3390/molecules28145469/s1, Figure S1: FT-IR spectrum of C12-En-C12 and C12-Bm-C12; Figure S2: 1H NMR spectrum of C12-E2-C12; Figure S3: 1H NMR spectrum of C12-E4-C12; Figure S4: 1H NMR spectrum of C12-E6-C12; Figure S5: 1H NMR spectrum of C12-B1-C12; Figure S6: 1H NMR spectrum of C12-B2-C12; Figure S7: 13C NMR spectrum of C12-E2-C12; Figure S8: 13C NMR spectrum of C12-E4-C12; Figure S9: 13C NMR spectrum of C12-E6-C12; Figure S10: 13C NMR spectrum of C12-B1-C12; Figure S11: 13C NMR spectrum of C12-B2-C12; Figure S12: Mass spectrum of C12-E2-C12; Figure S13: Mass spectrum of C12-E4-C12; Figure S14: mass spectrum of C12-E6-C12; Figure S15: Mass spectrum of C12-B1-C12; Figure S16: Mass spectrum of C12-B2-C12; Figure S17: Variation of specific conductivity with surfactant concentration C for C12-E4-C12 at 25 °C; Figure S18: Variation of specific conductivity with surfactant concentration C for C12-E6-C12 at 25 °C; Figure S19: Variation of specific conductivity with surfactant concentration C for C12-B1-C12 at 25 °C; Figure S20: Variation of specific conductivity with surfactant concentration C for C12-B2-C12 at 25 °C; Figure S21: Variation of specific conductivity with surfactant concentration C for DTAC at 25 °C.
