# Supplementary material for: Self-Aggregation, Antimicrobial Activity and Cytotoxicity of Ester-Bonded Gemini Quaternary Ammonium Salts: The Role of the Spacer
Source: Molecules. 2023 Jul 17;28(14):5469. doi: 10.3390/molecules28145469 (PMC10386392; doi:10.3390/molecules28145469)
Supplement: Supplementary file 1 [file molecules-28-05469-s001.zip › molecules-2471127-supplementary.pdf]

molecules

Self-aggregation, antimicrobial activity and cytotoxicity of ester-bonded gemini quaternary ammonium salts: The role of the spacer

Yaqin Liang*, Hui Li, Jiahui Ji, Jiayu Wang and Yujie Ji

Department of Chemistry, Changzhi University, Changzhi, Shanxi 046000, China
* Correspondence:liangyaqinfaye@126.com; Tel.:+86-0355-2178321

Figure legend

Figure S1: FT-IR spectrum of C_{12}-E_n-C_{12} and C_{12}-B_m-C_{12};

Figure S2: ^{1}H NMR spectrum of C_{12}-E_2-C_{12};

Figure S3: ^{1}H NMR spectrum of C_{12}-E_4-C_{12};

Figure S4: ^{1}H NMR spectrum of C_{12}-E_6-C_{12};

Figure S5: ^{1}H NMR spectrum of C_{12}-B_1-C_{12};

Figure S6: ^{1}H NMR spectrum of C_{12}-B_2-C_{12};

Figure S7: ^{13}C NMR spectrum of C_{12}-E_2-C_{12};

Figure S8: ^{13}C NMR spectrum of C_{12}-E_4-C_{12};

Figure S9: ^{13}C NMR spectrum of C_{12}-E_6-C_{12};

Figure S10: ^{13}C NMR spectrum of C_{12}-B_1-C_{12};

Figure S11: ^{13}C NMR spectrum of C_{12}-B_2-C_{12};

Figure S12: Mass spectrum of C_{12}-E_2-C_{12};

Figure S13: Mass spectrum of C_{12}-E_4-C_{12};

Figure S14: mass spectrum of C_{12}-E_6-C_{12};

Figure S15: Mass spectrum of C_{12}-B_1-C_{12};

Figure S16: Mass spectrum of C_{12}-B_2-C_{12};

Figure S17: Variation of specific conductivity with surfactant concentration C for C_{12}-E_4-C_{12} at 25°C;

Figure S18: Variation of specific conductivity with surfactant concentration C for C_{12}-E_6-C_{12} at 25°C;

Figure S19: Variation of specific conductivity with surfactant concentration C for C_{12}-B_1-C_{12} at 25°C;

Figure S20: Variation of specific conductivity with surfactant concentration C for C_{12}-B_2-C_{12} at 25°C;

Figure S21: Variation of specific conductivity with surfactant concentration C for DTAC at 25°C.

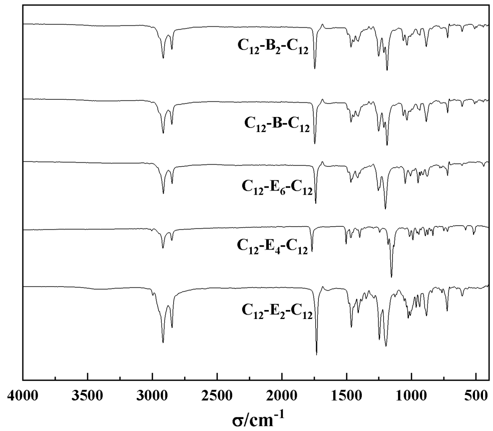

Figure S1 FT-IR spectrum of C_{12}-E_n-C_{12} and C_{12}-B_m-C_{12}

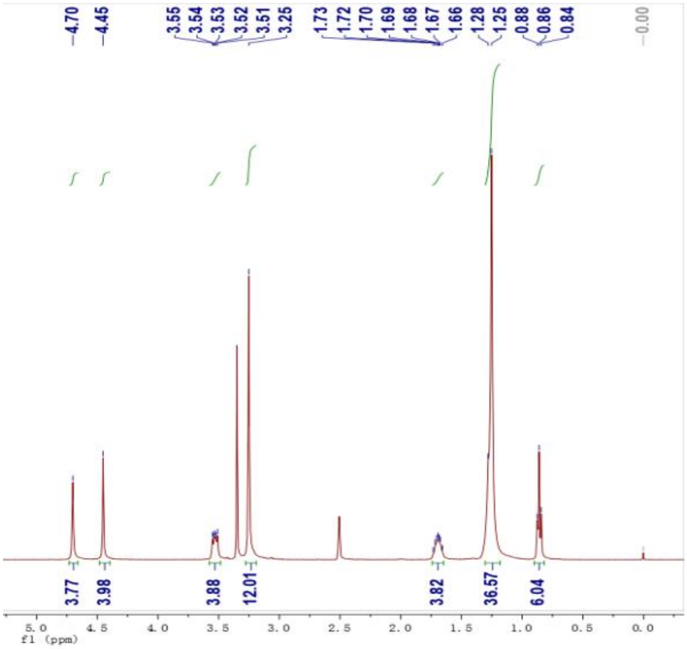

Figure S2 ^{1}H NMR spectrum of C_{12}-E_2-C_{12}

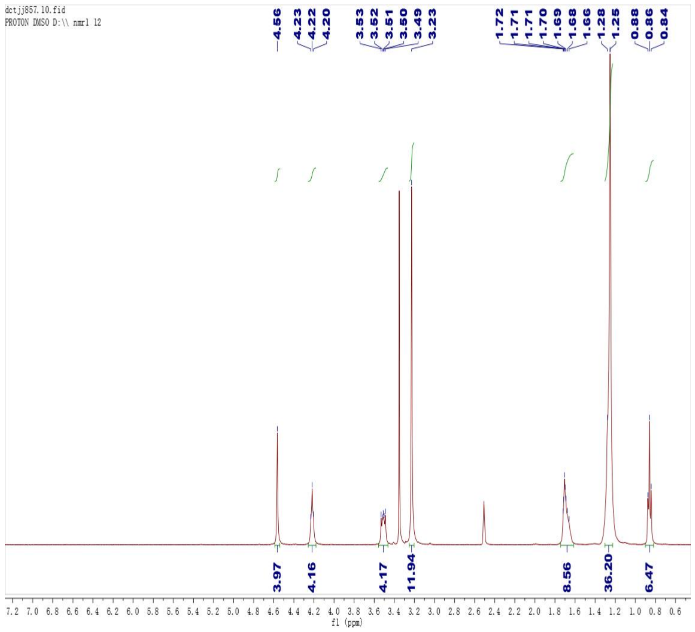

Figure S3 ^{1}H NMR spectrum of C_{12}-E_4-C_{12}

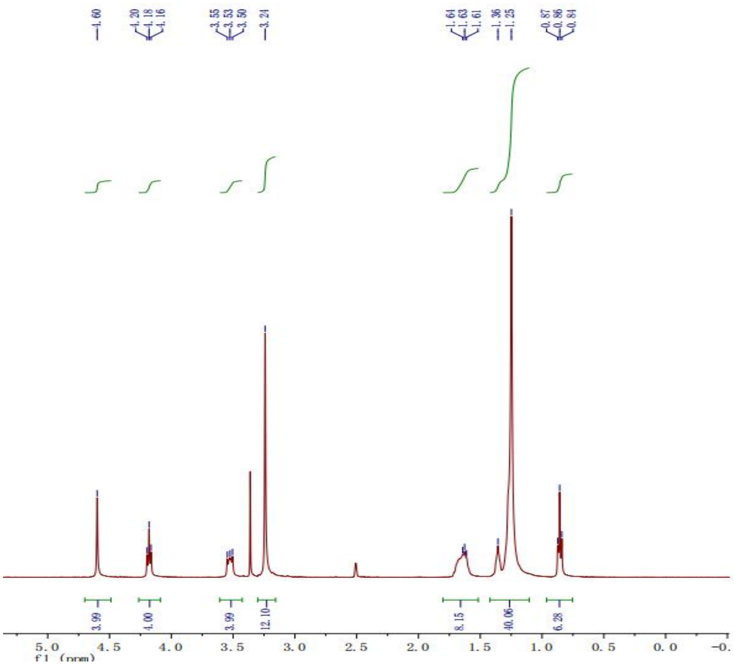

Figure S4 ^{1}H NMR spectrum of C_{12}-E_6-C_{12}

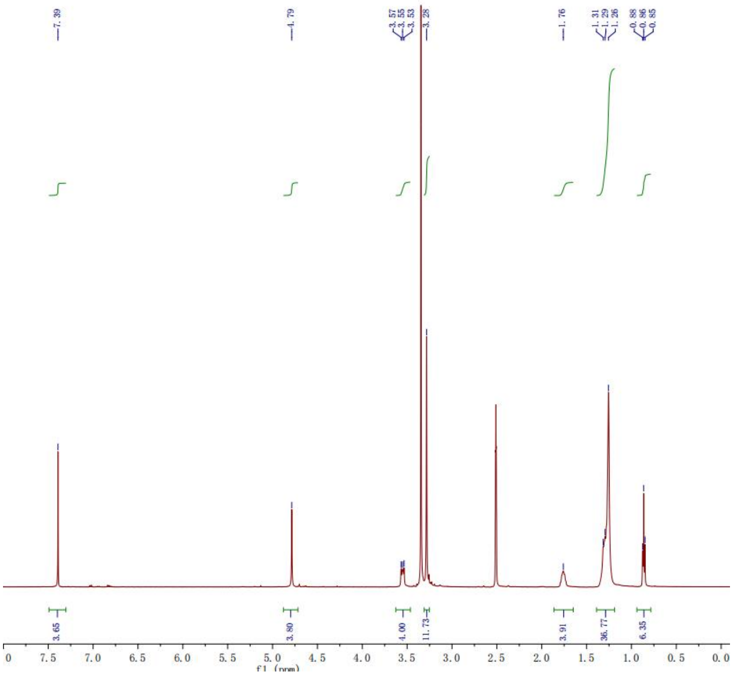

Figure S5 ^{1}H NMR spectrum of C_{12}-B_1-C_{12}

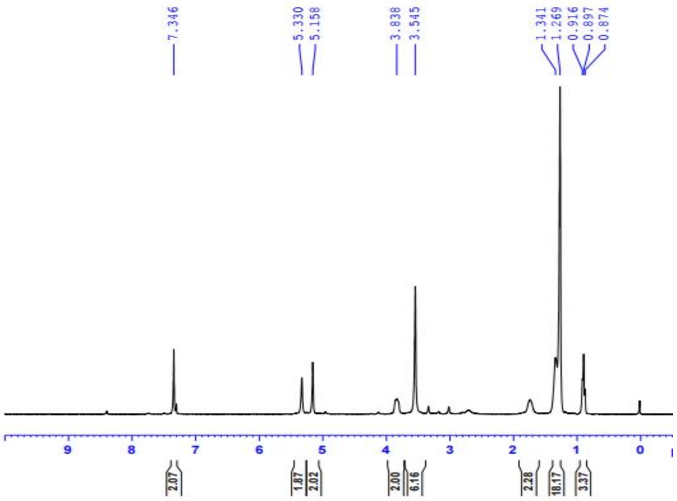

Figure S6 ^{1}H NMR spectrum of C_{12}-B_2-C_{12}

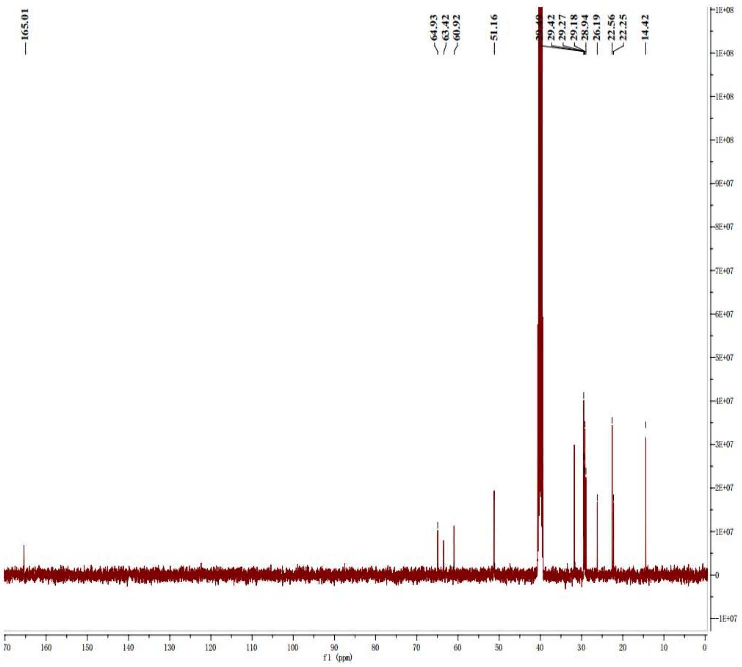

Figure S7 ^{13}C NMR spectrum of C_{12}-E_2-C_{12};

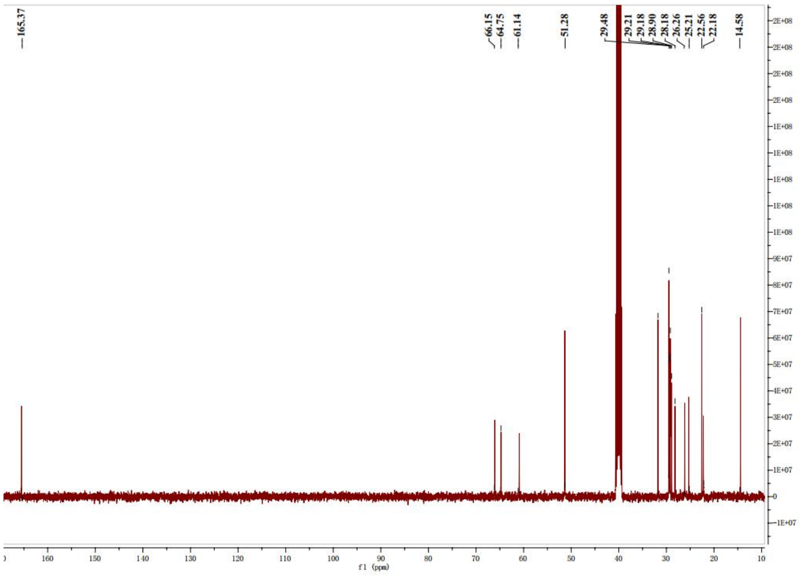

Figure S8 ^{13}C NMR spectrum of C_{12}-E_4-C_{12};

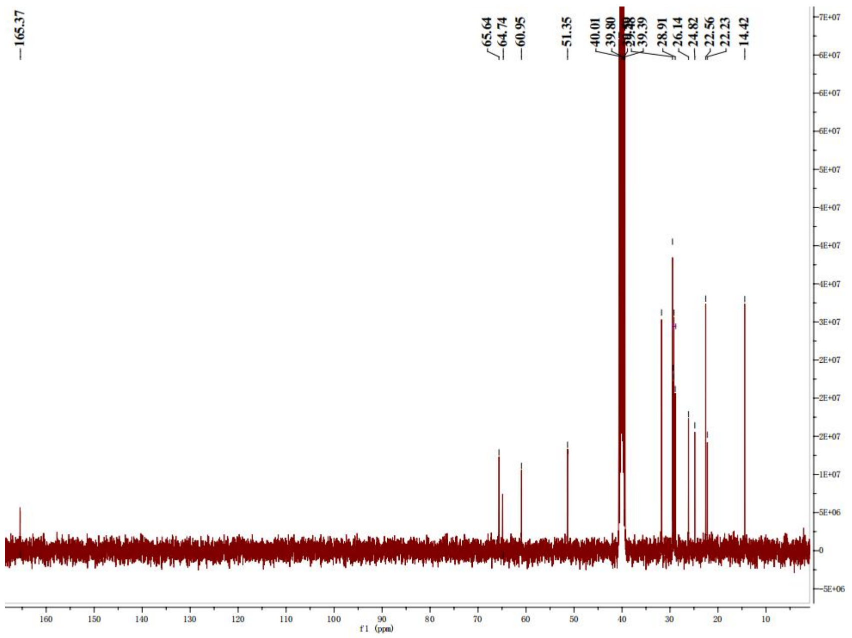

Figure S9 ^{13}C NMR spectrum of C_{12}-E_6-C_{12};

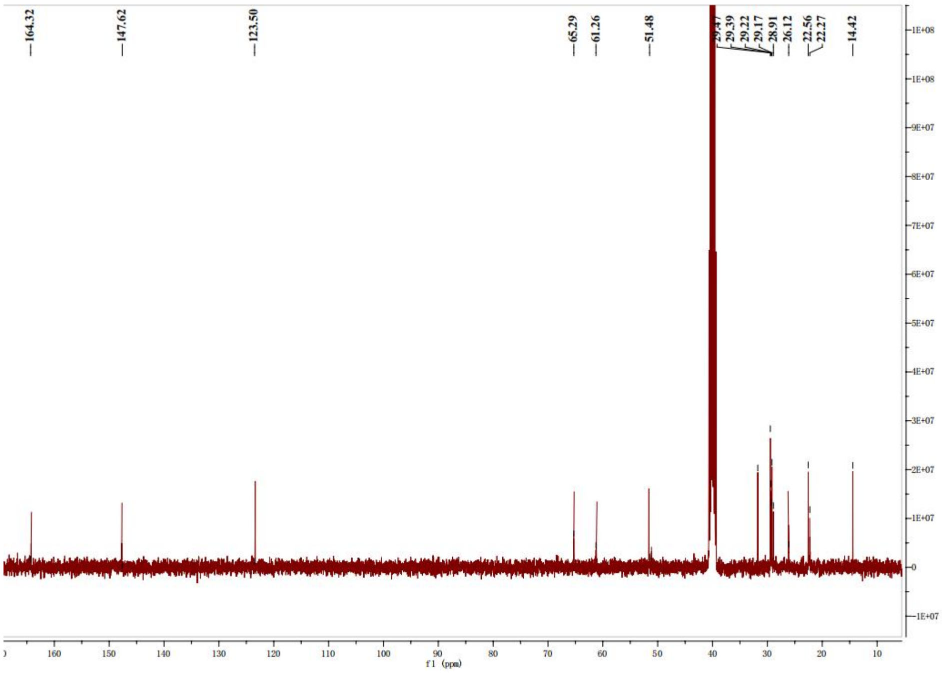

Figure S10 ^{13}C NMR spectrum of C_{12}-B_1-C_{12};

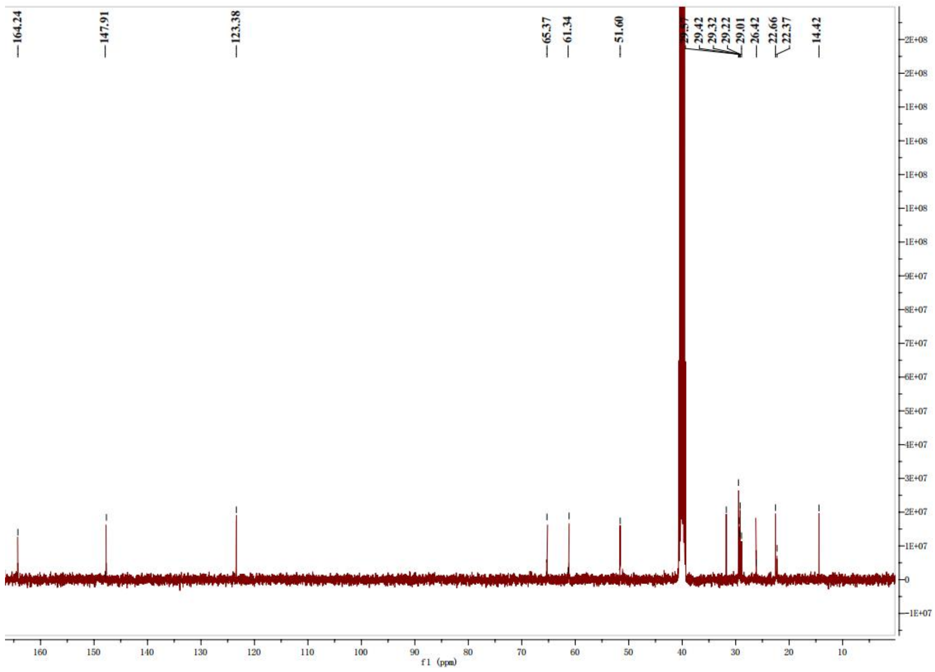

Figure S11 ^{13}C NMR spectrum of C_{12}-B_2-C_{12};

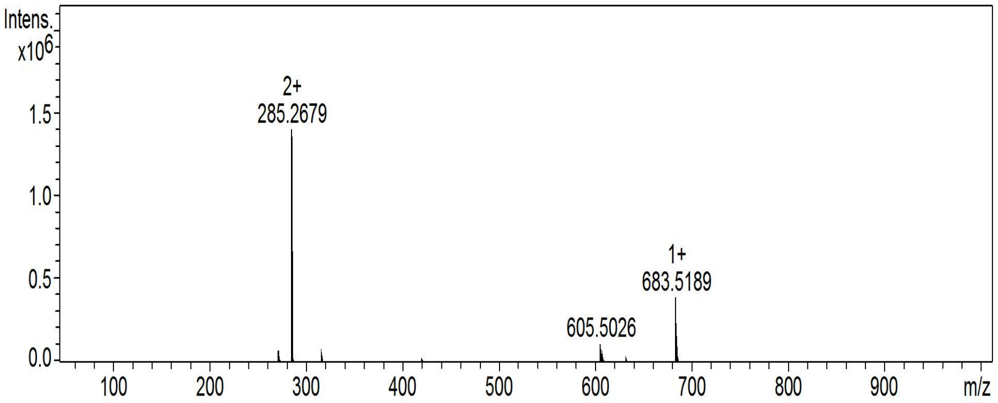

Figure S12 mass spectrum of C_{12}-E_2-C_{12}

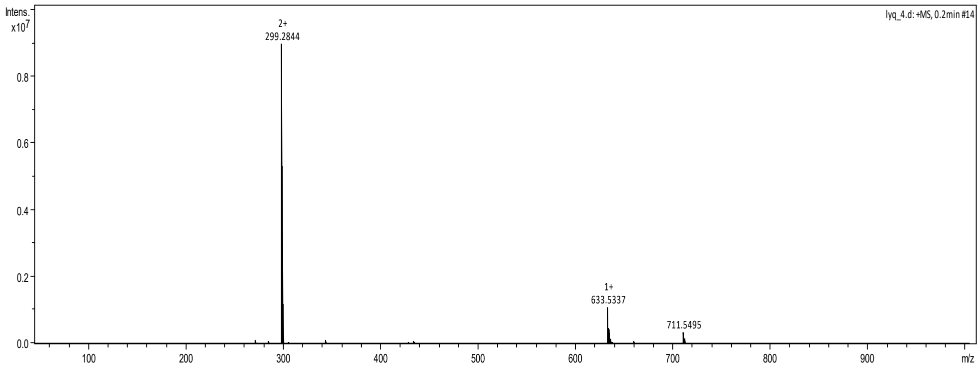

Figure S13 mass spectrum of C_{12}-E_4-C_{12}

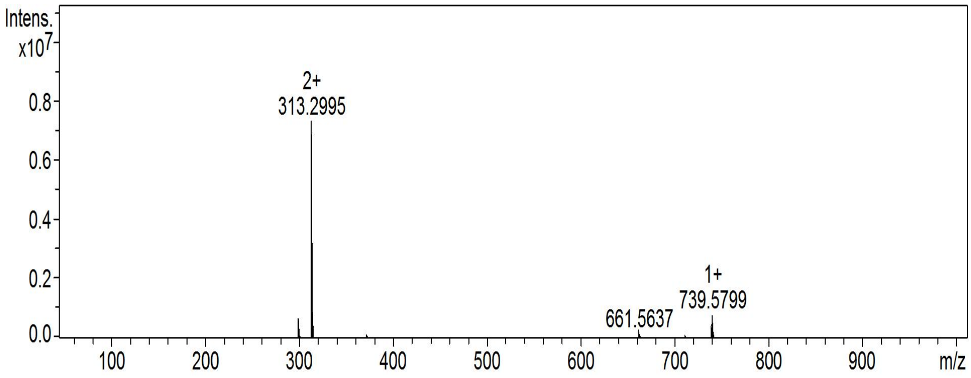

Figure S14 mass spectrum of C$_{12}$-E$_{6}$-C$_{12}$

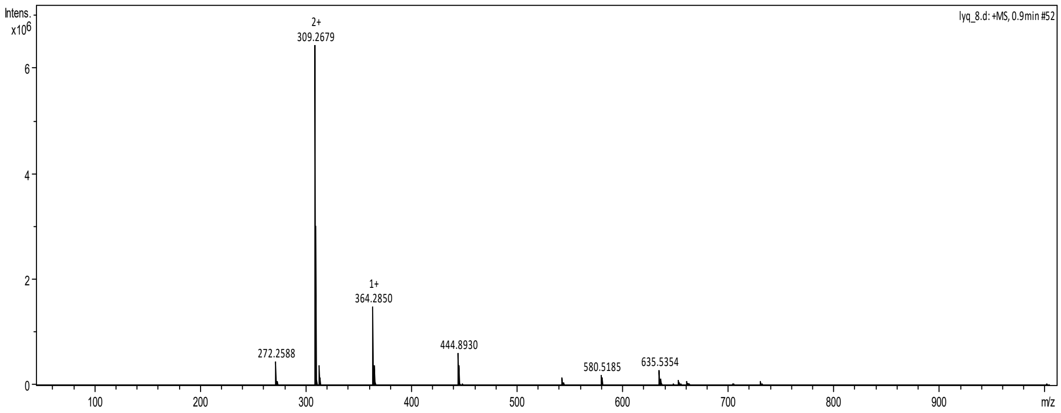

Figure S15 mass spectrum of C$_{12}$-B$_{1}$-C$_{12}$

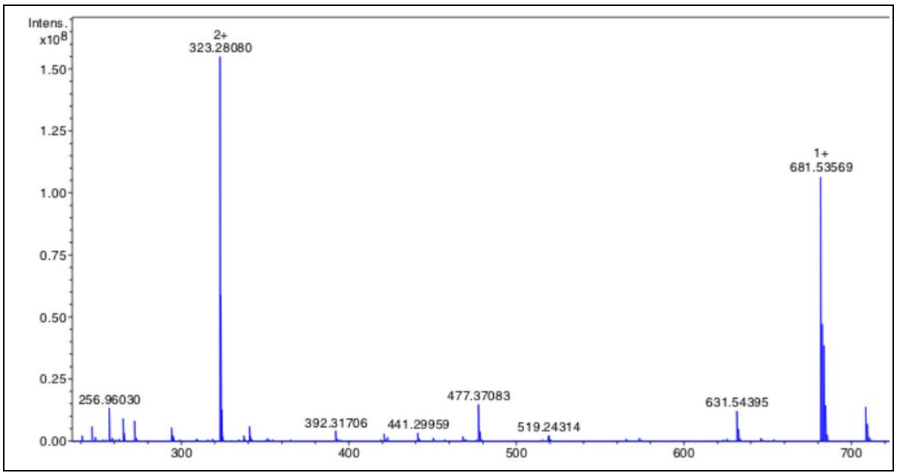

Figure S16 mass spectrum of C$_{12}$-B$_{2}$-C$_{12}$

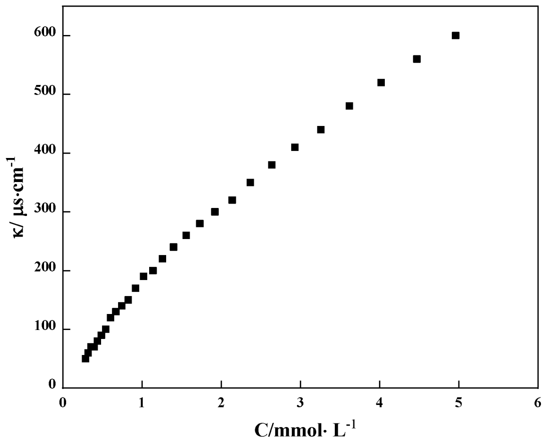

Figure S17 Variation of specific conductivity with surfactant concentration C for C_{12}-E_4-C_{12} at 25°C.

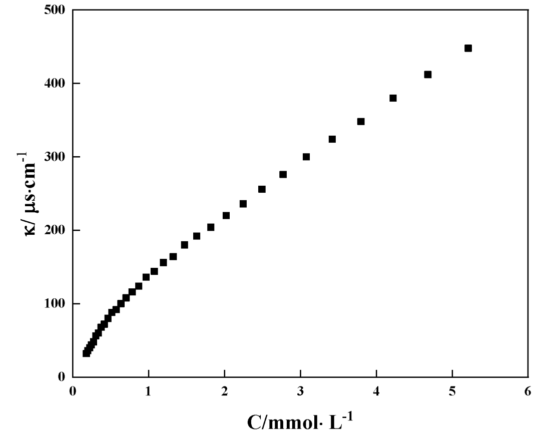

Figure S18 Variation of specific conductivity with surfactant concentration C for C_{12}-E_6-C_{12} at 25°C.

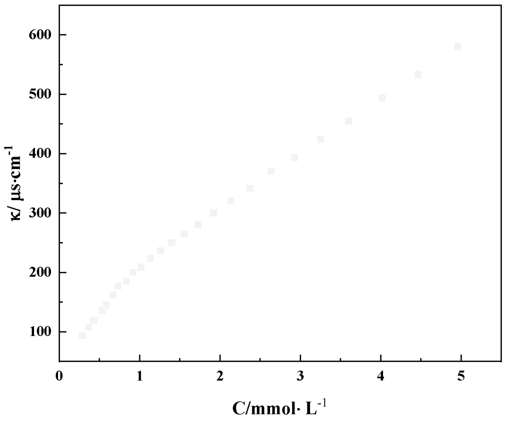

Figure S19 Variation of specific conductivity with surfactant concentration C for C_{12}-B_1-C_{12} at 25°C.

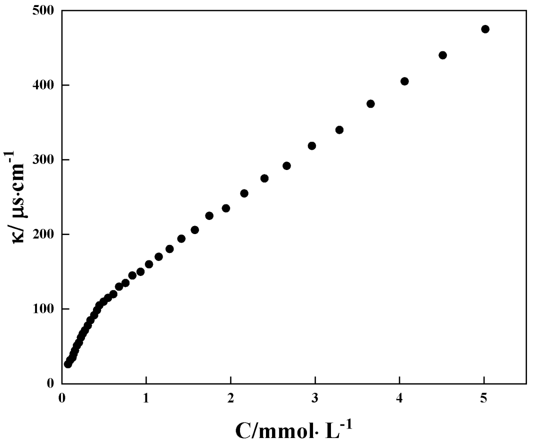

Figure S20 Variation of specific conductivity with surfactant concentration C for C_{12}-B_2-C_{12} at 25°C.

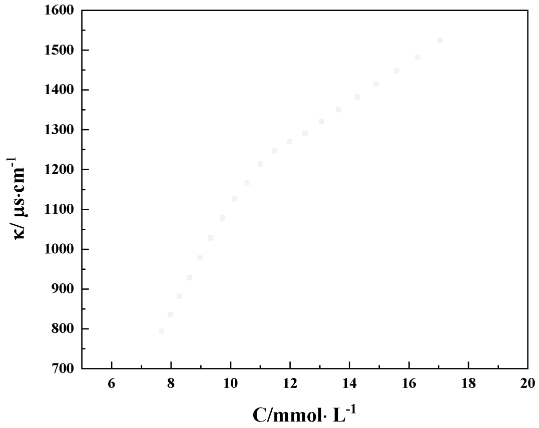

Figure S21 Variation of specific conductivity with surfactant concentration C for DTAC at 25°C.
